# Delayed Diagnosis of Temporomandibular Joint Dislocation in Severe Stroke Patients

**DOI:** 10.7759/cureus.68896

**Published:** 2024-09-07

**Authors:** Tatsuya Tanaka, Nobuaki Momozaki, Eiichiro Honda, Akira Matsuno

**Affiliations:** 1 Department of Neurosurgery, International University of Health and Welfare, Narita Hospital, Narita, JPN; 2 Department of Neurosurgery, Imari Arita Kyoritsu Hospital, Arita, JPN; 3 Department of Neurology, Shiroishi Kyoritsu Hospital, Shiroishi, JPN

**Keywords:** cerebrovascular disease, chronic temporomandibular joint dislocation, delayed diagnosis, dysphagia, manual reduction, neurological disease, oral feeding restoration, tracheostomy

## Abstract

A 79-year-old woman with a history of left cerebral infarction developed altered consciousness and left hemiplegia. CT of the head revealed a putaminal hemorrhage. She underwent tracheal intubation followed by a tracheostomy for long-term airway management. Despite improved consciousness, the patient continued to experience dysphagia and was fed via a nasal tube. Subsequent axial CT and 3D CT scans revealed an empty glenoid fossa in both temporomandibular joints (TMJs) with the condyles positioned anteriorly, consistent with chronic bilateral anterior TMJ dislocation. After an unsuccessful attempt at manual reduction, closed manual reduction was successfully performed under general anesthesia with muscle relaxants, allowing the patient to resume oral feeding. This case underscores the importance of considering TMJ dislocation in stroke patients with persistent dysphagia. Early diagnosis and timely intervention are crucial for improving patient outcomes in such cases.

## Introduction

Temporomandibular joint (TMJ) dislocation involves the displacement of the mandibular condyle from the mandibular fossa in the temporal bone. It is commonly caused by activities involving wide mouth opening, such as dental, intratracheal, and endoscopic procedures [1]. Neurological disorders, such as stroke, can also lead to TMJ dislocation [2]. While acute TMJ dislocation can be managed easily, chronic dislocation (lasting more than a month) is rare and challenging to treat [3, 4]. Herein, we report a case of delayed diagnosis of chronic TMJ dislocation associated with severe stroke in an elderly female.

## Case presentation

A 79-year-old female with a history of left cerebral infarction and hypertension presented to our hospital with impaired consciousness, a Glasgow Coma Scale (GCS) score of 8, right conjugate gaze deviation, and left hemiparesis. Upon arrival, her vital signs were as follows: heart rate 56 beats per minute, blood pressure 188/82 mmHg, respiratory rate 24 breaths per minute, SpO2 100% (with 5 L/min of oxygen via mask), and body temperature 36.5°C.

**Figure 1:**
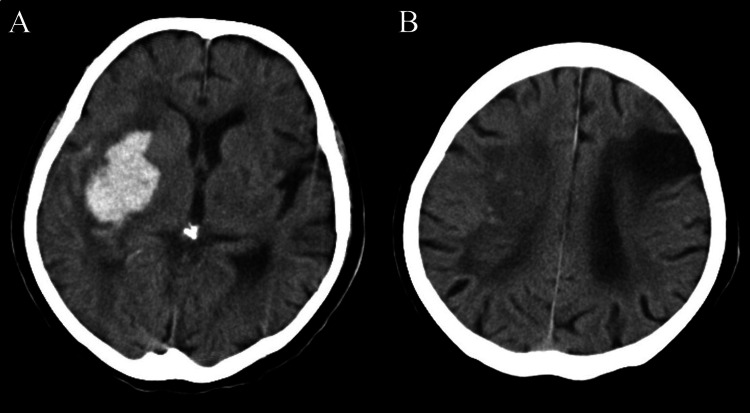
Initial head CT showing right putaminal hemorrhage (A) and chronic left cerebral infarction (B).

A head CT scan revealed an old infarction in the left frontal lobe and a hemorrhage in the right putamen (Figure 1).

Three-dimensional CT angiography (3DCTA) revealed no aneurysms or arteriovenous malformations. We initiated antihypertensive therapy with a continuous infusion of nicardipine and anti-edema therapy with an infusion of glyceol. On day 6, her GCS score decreased to 6, she developed obstructive breathing, her SpO2 decreased to 86% (despite a reservoir mask with 15 L/min of oxygen), and her body temperature rose to 38.6°C. Due to deteriorating respiratory status, endotracheal intubation was performed for airway management. Suspecting aspiration pneumonia, we administered ampicillin/sulbactam at 1.5 g four times daily. On day 20, a tracheostomy was performed. By day 56, her respiratory condition had stabilized, her GCS score improved to 4T6, and her level of consciousness had improved, allowing for tracheostomy closure.

**Figure 2:**
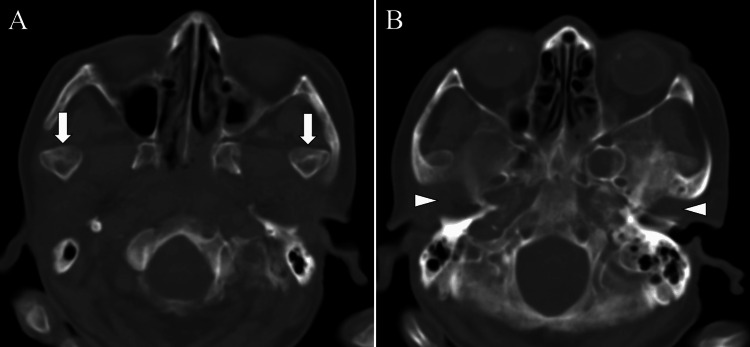
Head CT eight weeks after onset: CT revealing empty glenoid fossae in both temporomandibular joints (A; arrowheads) with anteriorly displaced condyles (B; arrows).

**Figure 3:**
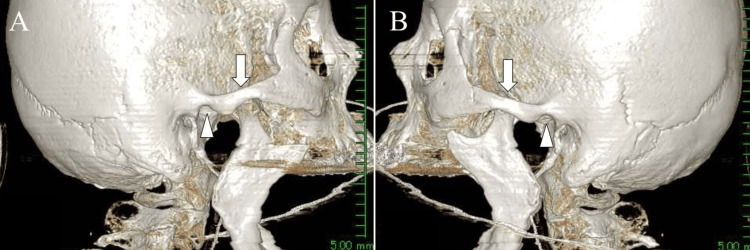
Three-dimensional CT eight weeks after onset: three-dimensional CT demonstrating empty glenoid fossae in both temporomandibular joints (arrowheads) with anteriorly displaced condyles (arrows). (A: right side, B: left side).

The patient had difficulty closing her mouth and exhibited dysphagia and dysarthria, necessitating enteral nutrition via a nasogastric tube. Subsequent axial and 3D CT imaging revealed empty glenoid fossae in both TMJs (Figures 2-3, arrows) with the condyles positioned anteriorly (Figures 2-3, arrows).

Accordingly, she was diagnosed with chronic bilateral anterior TMJ dislocation. Initial attempts at manual reduction and manual reduction under IV sedation were unsuccessful, so a non-invasive manual reduction under general anesthesia and muscle relaxants was performed. The reduction was successful, which allowed the patient to resume oral intake.

## Discussion

Chronic TMJ dislocation usually results from untreated or inadequately treated acute dislocation. Although rare, chronic TMJ dislocation associated with neurological disorders, including stroke, is prone to delayed diagnosis due to patients’ cognitive or neurological status and communication barriers [4-7]. Thus, TMJ dislocation should be suspected in severe stroke patients presenting with dysphagia. The muscles controlling mouth closure are innervated by the motor branches of the trigeminal nerve, and weakness of these muscles may contribute to TMJ dislocation in stroke patients [8]. The onset of TMJ dislocation can occur as early as 12 days after a stroke or as late as nine months, with a mean duration of 94.1 days; the reported incidence of stroke-associated TMJ dislocation is 0.25% per year [9].

Stroke is a major cause of dysphagia, occurring in 37%-78% of cases in the early stages post-stroke [10]. In our patient, the TMJ dislocation may have been induced by muscle hypotonia on the paralyzed side early in the acute phase, or by forceful mouth opening during intubation. Dysphagia was initially attributed to stroke-related paralysis, resulting in a delay of up to 8 weeks in recognizing the TMJ dislocation.

A prolonged interval between TMJ dislocation and diagnosis can lead to fibrotic changes in the soft tissues and muscle spasms, exacerbating the severity of the condition [11]. The longer the duration since the initial dislocation, the more complex the required procedures for reduction; nevertheless, management should initially involve non-invasive manual reduction [4-7, 12-14]. If this approach is unsuccessful, deep sedation or general anesthesia should be administered. Should these measures fail, invasive reduction techniques, including periosteal and muscular stripping, and traction using wires or other instruments, may be necessary. If these interventions are also ineffective, further invasive procedures, such as condylar removal, condylectomy, muscle incision, and/or the implantation of a TMJ prosthesis, should be considered [4-7, 12-14]. Post-reduction, the area should be supported with a bandage or similar device to prevent recurrence [13, 14]. In the present case, manual reduction and manual reduction under light intravenous sedation were unsuccessful, but non-invasive manual reduction under general anesthesia was successful.

It is noteworthy that in severe stroke patients, imprecise self-reporting during the treatment period may hinder the diagnosis of TMJ dislocation. Therefore, early detection of symptoms such as dysphagia, speech disorders, or difficulty closing the mouth is essential to prevent delayed diagnosis and facilitate effective treatment.

## Conclusions

This case highlights the need for careful recognition of symptoms of TMJ dislocation in severe stroke patients with persistent dysphagia. Although chronic TMJ dislocation is rare, it can lead to significant morbidity if not promptly diagnosed and treated. Recognition of symptoms, such as dysphagia, speech disorders, or difficulty closing the mouth, as well as early imaging and intervention, are essential to prevent long-term complications and improve outcomes in stroke patients.
